# What are the criteria for a good intervention study? Response: “Unrecognized ambiguities in validity of intervention research: an example on explicit phonics and text-centered teaching”

**DOI:** 10.3389/fpsyg.2015.00508

**Published:** 2015-05-05

**Authors:** Tom Nicholson, Laura Tse

**Affiliations:** ^1^Institute of Education, Massey UniversityAuckland, New Zealand; ^2^School of Curriculum and Pedagogy, The University of AucklandAuckland, New Zealand

**Keywords:** teaching reading, big books, explicit phonics, text-centered teaching, planned comparisons, *post-hoc* comparisons, validity, intervention research

Thompson (2015) has raised several validity issues about our study (Tse and Nicholson, 2014) while acknowledging that it would score well in terms of Troia's (1999) criteria for “What makes a good study?” A response to the critique is detailed briefly below.

Thompson's first point was that the study lacked evidence about what instruction children received prior to and concurrent with the intervention study. Interactions with teachers and the principals of the schools however indicated that reading instruction was similar from one school to the next. Any differences among schools and classrooms were also controlled for in that participants were randomly assigned to groups thus spreading possible effects of differences in instruction across all groups.

The second point was the absence of justification for the phonics rules taught however the article explained that the taught Anglo-Saxon decoding rules were from Calfee and Patrick's (1995) well-known explanation of Anglo-Saxon letter-sound patterns. The intervention followed their scope and sequence in the study. It is not clear why this might be a validity problem in that the study did reference the source of the phonics rules.

The third point was that participants' vocabulary age was low at 4.8 years compared with chronological age of 6.3 years and thus Big Books may have been inappropriate. Their standard score was 86 which is close to the average range (90–110) and there are studies to support Big Book reading with lower SES children such as these (Nicholson and Whyte, 1992; Valdez-Menchaca and Whitehurst, 1992; Whitehurst et al., 1994). The Big Books were also selected so as to be at the reading level of the children who were being taught and given that their reading level was in the beginner range the language should have been understandable for them.

The fourth point was that the article did not discuss whether children had opportunities to use their decoding skills to process the items of the pre and post-test measures. Although not reported our data did confirm that the combined group scored better on regular words (e.g., went) than irregular (e.g., love). The Bryant Test of Basic Decoding skills also gave opportunities to use decoding skills.

The fifth point was that the phonics group practiced phonics quizzes but the Big Book group did not practice reading of text. This was not completely the case. Children in the Big Book group did get opportunities to practice reading of text through the Big Book lessons. They did three readings of each text and read along with the teacher.

The sixth point was that the orthogonal analysis was not sufficient and needed to compare the performance gains of the combined group with those of the treatment control group (math-only). To do this however risked statistical error so instead of carrying out all possible comparisons among the four groups the decision was to use Helmert contrasts which were pre-planned orthogonal contrasts. This approach offered protection against statistical error (Kwon, 1996; Keppel and Wickens, 2004). As Kuehne (1993) has pointed out, using post-hoc comparisons increases the chance of type 1 error (in the study, to do six *post-hoc* comparisons across four groups would increase the possibility of type 1 error to 26%). The Helmert contrast procedure is common in other disciplines but less common in education. The way the Helmert contrasts worked in the study was that the control group mean was first compared with the overall mean score for the other three groups. Then the phonics enhanced Big Books group mean was compared with the overall mean for the two remaining groups (Big Book and phonics). Finally the means of the Big Book and phonics groups were compared. It was like peeling an onion. The logic was that if the control group was not better than the mean of the other three groups and if the phonics enhanced group was better than the mean of the combined Big Book and phonics groups, and if there was no difference in the contrast between the Big Book and phonics groups, then it can be inferred that the phonics enhanced group was superior to the other groups. The orthogonal contrast worked just as well as all possible contrasts with less risk of type 1 and 2 error.

The seventh issue was that speed of reading was not reported. Thompson's previous research would suggest a slower reading speed for the phonics enhanced Big Book group but it could counter-wise be argued that they would have gained similar fluency to the Big Book group because they also read Big Books. To answer this question, fluency would be a useful variable for future studies to find out which approach is more effective for fluency.

To conclude, one reviewer commented that the present study could be “a model for how such work might be conducted on a larger scale, which might lead New Zealand and other nations to progress in dealing with the [achievement] gap issue.” Replicating and scaling up the present study will clarify further whether enhancing Big Book reading with explicit phonics brings disadvantaged children closer to their expected reading and spelling age in a short time with only a small adjustment to Big Book instruction.

## Conflict of interest statement

The authors declare that the research was conducted in the absence of any commercial or financial relationships that could be construed as a potential conflict of interest.
